# The Passions
*"La Médecine des Passions; ou, Les Passions considerées dans leurs Rapports avec les Maladies, les Lois, et la Religion." Par J. B. F. Descuret. Paris. Second edition.


**Published:** 1850-04-01

**Authors:** 


### THE JOURNAL

OF

### PSYCHOLOGICAL MEDICINE

AND

### MENTAL PATHOLOGY.

APRIL 1, 1850.

### Art. I.—THE PASSIONS.*

Among the scientific works to which the competition for the Fothergillian medal of the "Medical Society of London" gave birth, was that by Dr. Falconer, of Bath, to whom John Coakley Lettsom presented the first medal in June, 1787,—its title, "On the Influence of the Passions upon the Disorders of the Body." Thus emotion was here represented as a *remedy*, presenting the bright side, or Utopia, of the passions.

Mons. Descuret, on the contrary, chiefly regards the term *passion* as indicative of *disorder*, translating the word παθος into *morbus*, rather than simply *affectus;* one very brief chapter being devoted to the salutary or remedial influence of the passions. His book, therefore, looks on the dark or clouded side of human nature—the Babel of the passions.

The primal essence of the passions seems to him to be an internal impetus,—instinct—want—appetite (*besoins*); the happy medium of its indulgence being the normal or healthy state of the mind. When some are in excess, or others in defect, or either in abuse, then a passion ensues. Thus he ingeniously essays to prove evil the abuse of good, vice the depravation of virtue—the passions thus resembling fallen angels. John Wesley had glanced at this in one of his sermons, we believe; and in later time, Spurzheim thus writes :—" L'affection ne serait qu'un mode de *qualité*, et passion un mode de *quantité*."

* "La Médecine des Passions; ou, Les Passions considerées dans leurs Rapports avec les Maladies, les Lois, et la Religion." Par J. B. F. Descuret. Paris. Second edition.

Hence our *wants*, animal, social, and intellectual, may be worked into *passions*, animal, social, and intellectual :—

"Les besoins animaux peuvent se rapporter aux instincts, les besoins sociaux aux sentiments, les besoins intellectuelles aux facultés de l'esprit. A ces trois classes de besoins, correspondent trois classes de passions, et trois classes de devoirs; des passions animales, des passions sociales, des passions intellectuelles; des devoirs animaux, des devoirs sociaux, des devoirs intellectuels."

The writer, while he thus shows himself sensible of the antagonism of man's nature, confesses the futility of attempting to obey to the letter the Delphic injunction, *γνωθι σεαυτον*, especially as regards the unfolding or analysis of the mystic union of soul and body. We may, however, all agree with this conclusion :—"L'âme est l'agent invisible dont notre corps revèle l'existence, comme Dieu est le créateur invisible, dont l'univers publie la force, l'intelligence, et l'amour."

That the mind is, indeed, a wondrous and an awful thing, none will gainsay who have deeply studied its phenomena—who have regarded the scope with which it conceives and accomplishes its intimacy with Deity or with Demon; the beauty with which its God had once endowed it; the depravity to which the wiles of Satan have reduced it; the thrill of happiness, or the agony of remorse, with which conscience, the essence of the soul, is blessed or agitated, as piety or sin have swayed its actions; and, above all, the final state to which it will be welcomed or doomed in its everlasting existence.

Since Plato discoursed on immortality, and deduced his chief argument, even for the existence of the Deity, from the nature of mind, the unfolding of its constitution and faculties have been discussed by philosophers of all ages—Malebranche, Descartes, Unser, Tillot, Hobbes, Locke, Hartley, Hume, Reid, Priestly, Stewart, Brown; while its intricate pathology has, to this moment, formed the deep study of many modern writers on insanity.

But the contrasted hypotheses of these learned sages, while they indicate the depth and sublimity of the subject, must yet impart a sense of humility, and make us feel, with Socrates, that we even now know little but our own ignorance.

Some of these ancients affirmed the soul to be a subtile matter composed of one or all of the elements: others believed in its immateriality. Thus Plato terms it *ουσια σωματος καὶ νοητη*, believing it not only mortal, but pre-existent; and Cicero, in his "Tusculan

Questions," writes—"Vim *divinam* mentis agnoscito." Plutarch also believed it an emanation of the Deity, and that the mind or understanding was the link that united it to the body. Aristotle believed the soul to be the substance of a body, *ψυχη ουσια σωματος τινος*. But we must check this discursion, merely adding, that when Virgil writes, "Mens agitat molem," he refers, not to the mind or soul, or anima mundi, but merely to the principle of vitality, which pervades the most wonderful system of our globe.

It is not, therefore, with the abstract metaphysics of the mind that we are about to treat. When we adopt the term "*moral* influence," we do not mean the decision of the mind to think this, to will that, or to act thus; but the physical influence, consequence, or product of its multiform associations (not only those excited by the senses, but also those of its internal workings or modifying of those impressions) on the health of the body.

The passions, like perturbed spirits, dragging reason from her path, the life of man becomes "une intelligence déchuë luttant contre des organes." How constantly, alas! is the organic stimulus triumphant, although the still small voice of conscience is as constantly whispering in our ear, in the words of Delavigne, "La vie est un combat dont la palme est aux cieux."

Now every sentiment must be, in multiform degrees, either pleasurable or painful. Yet so fine a line divides the sensations, that pleasure will often become pain in a moment: so the smile of the tickled child will turn to the convulsive sob, and the soft and delicate thrill of young affection become the *passion* of love.

Hence, as M. Descuret writes, "Toutes les affections vives, toutes les passions ayant le triste privilége de rendre le corps malade non moins que l'esprit."

In his allusions to the mutual influence of the brain and heart, through the sympathetic, the author seems to point to the heart as *the* seat of passion: "Les grandes pensées viennent du cœur;" yet he terms the brain the *creator* of images "qui vont aussitôt se reproduire sur les entrailles." Doubtless every emotion affects the heart's action, but thence the circulation of the brain is influenced, and this we believe is the real essence of the passion.

In our analysis of the *nature* of the passions, we might perhaps term emotion—sentiment—affection, *subjective* or individual; passion —*objective* or relative. There certainly appears, as we have long maintained, and as, indeed, Aristotle and St. Thomas Aquinas seem to have thought, a sort of antagonism or contrast of the affections of the mind, although still in intimate association. So every psychical bane may have its antidote—the casket of Hygeia *versus* the box of Pandora.

We sketch a crude arrangement of our proposition:—

| | |
|---|---|
| Anxiety | Resignation. |
| Vanity, pride, ambition, avarice | Wisdom, humility, devotion. |
| Deep attention | Mental repose, far niente. |
| Deep study | Recreation. |
| Morbid sensibility and irritability | Tranquillity, mental quietude. |
| Sympathy | Stoicism. |
| False delicacy | Simplicity. |
| Poetic frenzy—illusion | Reason, sacred truth, common sense, philosophy, mathematics. |
| Fear—apprehension, distrust, suspicion | Hope, confidence, boldness. |
| Timidity, cowardice | Courage, heroism. |
| Dread, terror, horror | Self-possession, analysis. |
| Sorrow, distress, grief, anguish, misery. | Joy, gladness, happiness. |
| Melancholy | Cheerfulness. |
| Chagrin | Satisfaction, delight. |
| Regret | Eumneia, happy memory. |
| Disappointment | Success. |
| Shame | Innocence, modesty. |
| Remorse | Good conscience. |
| Despair | Supreme bliss. |
| Anger | Placidity, self-control. |
| Envy | Charity, piety, benignity. |
| Jealousy, blighted passion | Happy love. |
| Hatred, disgust | Love (affection), admiration. |
| Misanthropy | Benevolence. |
| Revenge | Forgiveness, self-conquest. |

By studying more intimately, therefore, the morbid effect of thought and passion, we might often effect a salutary influence by establishing a train of contrasts in the mind. A perfectly healthy creature is so rare as almost to justify the axiom, "there is none sane but the Deity." If human life were for once "*mens sana in corpore sano*," existence itself would be a blessing. To live and breathe as in the elysium of the healthy child, were a paradise. As it is, health is most often essential to the exercise of what is termed good-nature, and is most intimately associated with that instinctive gratitude to God and love for all around which so often forms the basis even of practical Christianity.

But there *must be* a mixture of good and evil thought in the mind, which constitutes passion. To analyse and divide these has been the endeavour of psychologists from Pythagoras to Charles

Fourier. However opinions may be varied, there yet seems to be a leaning to the recognition of two principles or spirits in man's nature. Pythagoras, Plato, St. Augustin, Bacon, adopt this division, whether they be termed good and evil spirits, reasonable and unreasonable, superior and inferior, animal and social, cerebral and visceral or hepatic, or even a mental *duality*.

To the mind of Adam, created in purity and perfection, existence was but the blissful shadow of that paradise in which he was placed: every animal and organic function was pleasurable, for the goodness of the Creator thus first ordained that every function or action in obedience to appetite in his creatures should ever be associated with gratification: every thought even was a blessing. But this celestial and priceless gift was abused, and now pleasure is too often, like pain, destructive. Difficult and painful function might be once an *exception* to the rule, but progenial descent from morbid springs and through tainted systems has multiplied these exceptions *ad infinitum*.

We may believe, then, that Adam and Eve were in perfect happiness; but the long train of passions, from various causes, incessantly agitating the mind, are incompatible with pure and happy thought, while from its influence in producing or developing the ills that flesh is heir to, function is now an effort, a spasm, or a pang.

We write not thus in the spirit of complaint, to cast a gloomy shade over man's existence; the blessings of a wise and merciful Deity far exceed his inflictions. No, the priceless gift of immortality were worth, at least, an age of penance.

It was at the moment of Eve's transgression that we may believe disease and death to have been implanted: the germ, then in the course of development in the womb of Eve, would of course partake of her sinful nature. "I will greatly multiply thy sorrow and thy conception." And how instantaneously the influence of the wily serpent was evinced: the development of passions followed in rapid succession—indeed, they were almost synchronous. Shame and fear instantly prompted Adam to betray and impeach the beautiful being that was expressly made to be the solace and blessing of his bosom: "*She* gave me of the tree, and I did eat." Then came sorrow, regret, remorse, despair; aye, even before the expulsion from Eden. In process of time, the still more debasing passions swayed the heart of Eve's first-born, of which his brother was the victim—anger, envy, hatred, revenge, murder. Thus early was the dark development complete. As we contemplate the dawn of the mind, we see how nearly the series of passions which, from evidence of holy writ, we learn to have sprung up in the hearts of Adam, Eve, and Cain, resembles that now displayed in the child; fear and shame being the first evinced as the mind is unfolded.

The brain and nervous system are endowed with very contrasted degrees of excitability. A current will pass harmlessly through one mind, which may *destroy* another. This disposition is of course infinitely varied by circumstances. M. Descuret gives a very elaborate chapter comparing the influence of stations and professions, education, habits, &c. The tables attached are interesting, but somewhat speculative—we may, indeed, add unjust. Thus, among the vices of the physician, gluttony and incontinence are prominent! We have not thought of taking the guage of the moral scale of *French* doctors; but we were not before aware of our own propensity to cramming at the dinner-table: and unquestionably the study of anatomy, even waiving the important necessity of *character*, tends materially to subdue that romance and mystery which compose so much of the stimulus to licentiousness. These varieties of temperament are of the highest importance in our study and management of mental disorder—moral and medical remedy—converse, recreation, depletion, tonic, anodyne, are subjects intimately blended with the idiosyncrasy of the mind.

The subject of the Semeiology, or outward expression of the passions, is most interesting. Mons. Descuret's chapter especially refers to the two sciences, physiognomy and phrenology. The one, we may observe, is a retrospective, the other a prospective study, and both must be too frequently negative, that is, as far as the disciples of Lavater and Spurzheim are concerned. Physiognomy, or character of feature, may be a mere indication of what possibly *would be*, were there no control, or if circumstances arose to call into play; so also phrenology: both only evincing the disposition to a passion. But if, as students of Lebrun, we contemplate active physiognomy, we have a demonstration; the play of feature will, to the visus eruditus, be conclusive, speaking truth. Mons. Descuret's chapter is, however, ingenious on the subjects of hair, complexion, &c., although we think they would be chiefly valuable to a satellite of Vidocq, or a Newgate turnkey.

The progress of organic influences, we think, leads our author too far. The relativeness of passion and crime, and the treatment "medical, legislatif, et religieux," are, however, topics of much interest.

Anxiety is a universal feeling; it is the first that influences the mind of a child directly it begins to have wishes and hopes, and a consciousness that these hopes may not be fulfilled. Anxiety is thus a paradoxical combination of hope and fear, the preponderance, from the nature of the mind being in favour of the former.

Anxiety is the dread of something worse than the present. In the moments of success, of course there is a thrill of delight, but this is often evanescent as the lightning flash. Its objects are various; some may be justly termed moral anxiety, as that of a wife for her husband, a mother for her child: in them there is a holiness which excites the deepest sympathy. Others have a more impure source, as pride, avarice, envy. To a heart thus beset, there is no rest; the passions are never satisfied, enough (of honour or of wealth) is a term absolutely indefinite. These feelings, which Mons. Descuret discusses as separate passions, are but different phases of anxiety. Pride, arrogance, ambition, are but a selfish desire to be raised above others in the world.

Vanity, the pride of woman, is an anxiety to surpass others in beauty, to be admired. Eccentricity, as of the Quaker and beggar, is but a slighter form of vanity. "I see your vanity," said Socrates to Diogenes, "in the holes of your coat, in your rags." How essential is it to check these sentiments in the bud, by speaking truth and avoiding flattery to childhood; when we know how perilous are these grand errors of the mind even to fatality. Confirmed pride, and vanity wounded or curbed by real superiority, may end in death. The pride of Cain murdered his brother; the wounded pride of John Keats was a fatal shock to his own life. Would that the Preacher could convince us all of the littleness of worldly thoughts, and turn them to futurity and heaven; then pride and vanity would live but as shadows of the past, and we should exclaim:—

"Why all this toil for triumph of an hour?"

Emulation is the anxiety to excel by noble effort; ambition, rather to be installed in the seat of honour, no matter how. "Celle-ci," writes Mons. Descuret, "est crime, l'autre est vertu."

The list with which we are presented of the victims of ambition, by murder, execution, and suicide, is somewhat strained. Kings, whether ambitious or not, are *ever* exposed to the assassin. Brutus and Cato were stoics, and made a monstrous merit of leaping prematurely out of life. Christianity, however, has put Stoicism to flight, and now we abhor the act of suicide, even though, like that of Curtius, it were to save our country.

Apropos of suicide, we remember somewhere to have read a chapter beginning thus—"In the gloomy month of November, when Englishmen cut their throats." In Mons. Descuret, however, we read this—"Cheyne rapporte qu'en Angleterre l'automne et les vents d'ouest sont féconds en suicides, le professeur Osiander dans le nord et l'Allemagne partage cette opinion; Cabanis et Esquirol ont aussi observé, que le passage d'un été sec à une automne humide est plus favorable au développement des affections abdominales, dont le suicide depend assez souvent."

The fate of the Presidents of the Convention is very impressive; for this and other tables of much interest we must refer to the book.

At the shrine of ambition a man thus sacrifices not only the holier thoughts of his soul, that might have ensured his passport to the gates of heaven, but wrecks even his earthly happiness. The pride of success soon palls upon the sense, the whisper of adulation only incites to further, often to vain, struggles to ensure a repetition; and when all earthly grandeur and power are at length attained, the proud and anxious possessor stalks through his painted halls, fumbles his jewels and his crosses, and then looks out on his broad lands and frowning forests, and deplores (that is, if he can moralize) that his heart is not capacious enough to enjoy them according to their splendour or their magnitude.

And the passion of gaming! How epigrammatically are its consequences expressed in this inscription for the front of a "hell"—

"Ici deux portes à cet antre:

L'une s'ouvre à l'espoir, l'autre au crime, à la mort;

C'est par la première qu'on entre,

Et par la seconde qu'on sort."

On its physical effects we will quote a passage from our author:

"L'infamie n'est pas la seule terminaison de cette passion funeste: on la voit encore très communément finir par la misère et la mélancholie, quelquefois par la folie, le meurtre, et la suicide! Les joueurs étaient fort sujets aux engorgements, des viscères abdominaux, ainsi qu'aux affections anéurysmales du cœur, ou de la crosse de l'aorte."

'Tis pitiful, indeed, that man should thus pervert his intellect, and hug the scorpion to his breast. 'Tis pitiful to know how vain will be our efforts to check the headlong course of one on whom the monomania of gaming, the "manie du jeu," has taken so deep a hold,—to know that while the health of the body is sapped, the brain may soon become the source or seat of drivelling or raving mania, when it may be *too late* to avert the awful peril of the immortal spirit.

Regarding the comparative or national propensity to play, Mons. Descuret thus writes: — "On pouvait classer les joueurs passionés dans l'ordre suivant: Chinois, Anglais et Anglo-Américains, Italiens, Espagnols, Russes, Allemands, Polonais, Belges et Hollandais, enfin les Français les moins acharnés de tous." This *aviditas æris* or *aværis*, which is whimsically cited as the origin of the term avarice, is one of the worst forms of idolatry. The "good old gentlemanly vice" not only keeps wealth from the community, but the services of its slave.

That it is often a symptom of disorder, we do not doubt, and we refer, *en passant*, to the case of a lady, recorded by Alebert, to illustrate this. "Cette dame, vaporeuse et mélancholique pendant six mois de l'année n'usait alors de ses revenus, qui étaient considérables, qu'avec une parcimonie sordide; mais elle se faisait admirer par une générosité sans bourne aussitôt qu'elle était revenue à son état normal de santé."

To the slaves of pleasure, the devotees of Bacchus and of Venus, anxiety is ever an attendant demon, except in the moments when intense excitement drowns the heart and mind in voluptuousness. The former must not be limited to those who indulge in *alcoholic* drink. The prohibition of wine by the prophet removes not the stain of intoxication from his proselytes. Indulgence in the *mechish* of Stamboul, or the *bouang* or *pust* of Ispahan, reduces the voluptuous slave to a state as abject as that of the drunkard of our own nation. It is, however, rather a check to *sexual* passion. The abuse of the grape is constantly followed by the abuse of physical love. To

"Sine Baccho—friget Venus,"

add

"Baccho urit Venus."

But it is not the influence of vicious or unholy passion alone that engenders disquietude of mind. Intensity of study, within certain limits a recreation, is rendered, like excess of physical pleasure, the baneful source of disease.

Mons. Descuret terms it "*Manie d'étude excessif;*" and we agree with him, especially when a life is devoted to the attainment of a world of *profitless* learning—the recluse student shutting himself up, like a grub in a shell, and the mine of wealth, which he had accumulated, dying with him. Of such a being we have a long and interesting history in the biography of the Hungarian Montelli. As to its individual influence, remember Ariosto, Collins, Cowper, White,

Byron, Coleridge, Malibran, Paganini. What wonder that the mighty power of their genius, the divine inspiration, the "*don du Ciel*," should thus run wild until the lamp of life is exhausted. The fascination of poesy is, alas! too often a meteor which blazes but to die.

> "Look on me, there is an order
> Of mortals on the earth, who do become
> Old in their youth, and die ere middle age,
> Without the violence of warlike death:
> Some perishing—of study,
> And some insanity."*

How often, also, does the creation of a beau-ideal of the thought render the mind deeply hypercritical, dissatisfied with all but perfection, (as the girl who died for love of Apollo's statue;) and the body so acutely sensitive as almost to "die of a rose in aromatic pain." What writes Wordsworth? and what but the unconscious prophecy of his own fate was the apostrophe of Byron on "unhappy White?" The poet's eye, like the Titan, would scale Olympus; like Semele, would look on Jupiter in all his glory, and then perish. Study in this excess is all-absorbing, and becomes, as Paganini confessed, a "consuming passion." While our listening ears hung in ecstasy on the magic of the maestro's wondrous art, his brain was lighted up with preternatural fire, with more than fever heat; and then came that intense irritability, which not only rendered a discordant whisper agony to his sense, but well nigh banished sleep from his pillow.

This, then, is the penalty of genius. Thus does the Creator confer on man that exquisite balance of enjoyment which ordains that what it gains in intensity is lost in duration—that pleasure heightened by excesses should be followed by depression and by pain.

And what the prophylaxis? Simply repose, the fallow of the intellectual brain; during which, the unstrung bow might regain its elasticity. But the restless spirit will not submit to this abeyançe of genius; it will fly, perchance, to the baneful solace of opium or alcohol; for it is not every genius that can soothe the brain with Newton's weed or Byron's dose of salts.

Then (if we may presume to make a digression on sleep, as old Burton writes, that "chiefest thing in all physic," and which Paracelsus eulogizes as "omnia arcana gemmarum superans et metallorum") would it not be somewhat paradoxical to expect a *genius* to sleep soundly and well?

* Manfred.

> "My slumbers, *if* I slumber, are not sleep,
> But a continuance of enduring thought,
> Which then I can resist not: in my heart
> There is a vigil, and these eyes but close
> To look within."*

It might be, if we were gifted with the power of working with one hemisphere or one brain, as the mechanic with one hand, that we might hope for the slumber of one mind while the other was at work. It is not so; nor is there any royal road to the cell of Morpheus, however a late enthusiast of the name of Gardner vaunted to this end his science of *hypnology.* The principle of his system was *monotony,* and consisted chiefly in *listening to the breathing;* but we proved it to be a fallacy, although Gardner obtained the assurances of Sheridan Knowles, and other sons of genius, of its truth.

No; the curb of imagination must be applied *early,* ere it has drunk in its excess of sunlight: ere it has become enslaved by its idol worship, it should be taught the beauty and utility of truth. The very thoughts of childhood are in themselves a romance; and without the light of reason, then but just dawning, and with the opening passions incessantly swaying the heart, it may be very difficult to turn to the good and eschew the evil, to prevent the establishment of *erroneous motives* of action.

It is in early youth that the mind is allowed to *dream* while it should be instructed; and in the visions of imagination too often arise ideas frivolous and useless, perchance sinful, which become the idols of our thought, the food of our life.

Then when the youthful sentimentalist begins to bear his part amid the stern realities of life, he awakens from his dream, and discovers that he has not only to discipline his mind afresh, but to *unlearn* all that romance had taught him; and in this struggle health is endangered, nay, the mind itself may yield. How difficult the contention none can tell but those who have endured and survived the struggle.

Envy and jealousy are anxiety of the most debased kind—the desire to reduce others below our own level. But it is a bad marksman, this envy; it aims at others, but often hits itself: the story of Haman and Mordecai, for example. M. Descuret has collected, in illustration of this, (as, indeed, of all his subjects,) many very interesting and novel histories; one especially so, as recording the development of cancerous disease by a fit of jealousy. We fre-

* Manfred.

quently observe, too, the lighting up of tubercular consumption by long anxiety.

The feeling of anxiety is, indeed, one continued heartache—more poignant than sorrow or grief, those pains of memory that know the worst, and, by the mind's elasticity, may soon be dislodged. The usual effect of anxiety is low nervous fever and venous congestion. The secretions become diminished in quantity, or unhealthy and depraved in quality; for so surely as the enlivening passions oxygenate the blood, do the depressing feelings accumulate carbon. By this poison a constant reaction is going on, by which the original malady is wofully increased. Thus is established a sad train of neuroses, neuralgia, melancholy, hysteria. If the brain be long oppressed by its poison-blood, we may ever fear the climax of tædium vitæ—suicide; for there is a state of universal *malaise*—all is going wrong: digestion, assimilation, circulation—the chief agents of the vital principle—fail; absorption of fat succeeds, and atrophy ensues. The secretion of milk in the anxious mother is checked or depraved, and by this half-poisonous fluid are many of the gastric and convulsive diseases of early infancy induced.

The system thus reduced is intensely susceptible of malaria and other infectious agents. On this point, regarding cholera and plague, are recorded many interesting stories.

The combination of anxiety and forethought constitutes fear or dread: its more intense degrees, though not essentially prospective, are terror, dismay, horror. They arise from the belief that something will occur to injure materially our present condition.

During the first impulse of sudden fear or fright, the pulse is often bounding; but during protracted fear, its force will become diminished and variable. The circulation is sluggish: it may be so complete a remora in the large trunks, that the skin becomes pallid, ashy, or dusky, and shrunk; the effort to recover from this state being attended by rigor—the shivering of fright. The capillaries and exhalents may still be relaxed, and then there is the cold, clammy sweat, so analogous to the colliquative oozing of the morient collapse. Hence, also, the occurrence of diarrhœa—sometimes of diuresis; the sphincters being not only enervated, but highly irritable, a very minute quantity of water inducing a desire of micturition. On these points, Bonetus, Pechlin, and others, relate very curious though often indelicate stories.

Under an intense degree of dread we observe a suspension of volition; the motive power is paralysed; tremors affect the limbs, and the senses, especially that of sight, are often in abeyance; and if this continue, it may end in apoplexy and sudden death.

The secretory vessels partake of this atony; that of the arteries of the piliferous bulb is evinced by the sudden turning grey of the hair, of which we have ourselves known instances. Others are related by Scaliger, Boulli, Donatus, Holland, &c. M. Descuret relates a curious story of a young Sardinian, whose hair turned from raven black to snow white in a few minutes. He was, while suspended on a rope, attacked by eagles, whose nest he had robbed of the eaglets. In defending himself, with his knife he cut the rope *half through*, and although nearly dead with terror, was drawn up in safety. This effect will sometimes be checked by the removal of the cause of fear, the hair gradually assuming its former tint. In like manner, sexual passion may subside during the prevalence of fear.

Fear, induced by threat of penalty, may often prove remedial when medicine has failed; especially in cases of epidemic or imitative monomania, as in the leaping ague, the dance of the middle ages, &c.; and the curious suicidal monomania of a bevy of the holy sisterhood, which was directly checked by threat of exposure of their naked bodies after the commission of the crime. Fear may be also beneficially excited in disorders marked by clonic spasm, in the reduction of hernia, and of dislocations in which our common efforts are thwarted.

In the more intense sensations of dread, terror, and horror, the systematic tumult is of course greater. There is often lividity or turgescence of the face, throbbing and distention of heart and arteries, and convulsion, and also a contraction of the integument, which will cause the hair to *stand on end.*

The effect of dread is sometimes displayed in the induction of the very disorder which is feared; the symptoms of rabies, for instance, from the extreme fear of having been bitten by a mad dog. For very curious illustrations, we refer our readers especially to Sir Alexander Crichton's work, to Pechlin, and our friend Dr. George Moore.

The more prosperous influence of terror is evinced in the sudden scaring away of neuralgia, toothache, and epilepsy, often transiently, but sometimes permanently.

If we may believe Pausanias and Herodotus, Battus and the son of Crœsus were in a moment restored to speech—the one at the sight of a lion, the other when a soldier at the siege of Sardis was about to kill his father. Dr. Wigan relates a parallel case of the son of an export merchant in the City, in peril of drowning in the Thames.

To the influence of terror also may be ascribed the wondrous efficacy of *charms;* the rubbing of a wen with the hand of a suspended criminal, the wearing of toads and lizards round the neck, drinking the blood of a dying gladiator, &c. &c. When terror is combined with present danger, we see the courage of despair; a dilemma of terror imparting often an almost supernatural power. Such straining, however, will often induce apoplexia or mania in one subject; in another, suspension of the heart's action and sudden death.

The essentially depressing emotions are sorrow, grief, anguish; passions which tend to soften the heart, if pious resignation be their handmaid. Melancholy, chagrin, despair, remorse, are feelings of a darker hue, and prove a cankerworm to the heart and mind. These passions are of course retrospective, and refer to something which *has been.*

The corporeal effects of sorrow, grief, regret, and anguish, resemble those of fear—the remora of the black blood inducing lassitude, debility, despondency, and inaptitude to action. The engorgement of the heart may cause its flaccid or attenuated parietes even to yield, by inducing unnatural efforts. The constant expression of grief is, that the heart seems as it would burst. To relieve this oppression, we have the instinctive deep sigh and sobbing depending on convulsion of the diaphragm.

The intense degree of grief is all-absorbing. The mind broods in silence over its woes, and so reluctant is it to admit an intrusive subject, that it is annoyed both by the conversation of friends, and even by impressions on the senses. They who deeply mourn, therefore, retire into lonely and quiet seclusion, and there they nurse their sorrows, jealous of all intrusion. And now may be lighted up a train of feelings as distressing as they are obnoxious to remedy. Indeed, the languor and apathy of melancholy ever indispose to that which might effect its cure. Exercise might restore health to the blood, remove dyspepsia, acidity, and gastrodynia, and society might displace the spectres of the mind by happier and healthier thoughts. In sensitive girls, the state will be one of hysteric melancholy—its symptoms often assimilating almost every painful affection, leading the physician even to extreme errors of treatment. The hepatic system is usually deranged, the bile black and viscid, sometimes pitchy, the origin, indeed, of the term: hence assimilation must be defective.

Thus overshadowed, how can life be happy?—the hours pass heavily, the heart hath no joy, no change of scene yields relief, unless that last awful change, a self-sought passage to the grave.

Melancholy thus grows by what it feeds on, yet how often has the physician disregarded the principle of cheerful association, and let the judgment on the melancholic go by default. Society and sympathy might become the life itself of the sensitive heart, that without it will droop and decay. The savage, indeed, may roam in a desert uninfluenced by its desolation; he has become familiar with solitude, has made it a world of his own. The solitude of the social being is too often peopled with spectres, especially if love be debarred access to the sufferer, who soon feels that his place in the busy world is vacant. His life is a blank. This is not yet the darkest picture: where irreligion has marked a life, every thought is a sting, and day and night, a fury haunts every waking moment and every agitated dream. From such a mind, derangement cannot long keep aloof, the degrees and forms of mania depending mainly on disposition, temperament, sex, and age.

The longing after home is a form of melancholy which often induces acute disorder, "Maladie du pays," unless the desire be gratified. The spring of this "nostalgia" may be no more than a few melodious notes: the pibroch and the rans de vaches have so excited this intense longing, that the national airs have been forbidden in the regimental bands during cantonments in foreign climes. This longing will also go far to induce a train of more formidable and loathsome symptoms in the African slave—"Cachexia Africana."

How important, then, is it to penetrate and analyse the secrets of the mind; in this often rests our chief hope of ministration.

But the same influence will sometimes be itself remedial. Our late friend, David Uwins, alludes to the case of a maniac officer in whom deep madness was cured by the notes of a shrill pipe, which, exciting pleasing reminiscences in his brain, put the whole system in a healthy course, and recovery speedily ensued. It requires, however, some address to conceal the *motive* of such a mode carefully from the maniac: if his *cunning* discover the plot, our effort will be thwarted.

The debasing emotions that have more especial reference to our social state, form one dark family of passions; yet all may be passive save one, to which, however, all the others too frequently lead. Of envy, hatred, jealousy, anger, the climax is, alas! most frequently *revenge*. Hatred, envy, and jealousy, when they sit and brood over their ills, often prove merely depressive, and, indeed, may be termed own sisters of melancholy; they are self-tormentors. Envy especially, would lose one eye to ensure the loss of both to the object of his passion, and like Haman, he is often foiled with his own weapons. Anger at once *excites* the system. Horace terms it "brevis furor;" Charron, "chemin à la manie;" and our popular synonyme is "being in a *passion.*" Mons. Descuret alludes to it as "un besoin excessif de réaction."

If unrestrained, the head becomes heated, the veins swell, the eyeballs flash with unwonted fire, and seem prominent, and the muscles are excited involuntarily to unnatural action. If protracted, the liver is gorged, the stomach and colon loaded, and the end may be apoplexy—convulsion—mania.

Mons. Descuret refers to two sorts of anger—"colère rouge, or expansive, and colère pale, or spasmodic," and offers some very useful precepts for self-government and the moral management of children. On the latter point, he says anger is constantly induced by our allowing children to *have what they cry for.* But we were not before aware of the special influence of cold and heat in exciting or subduing *national* anger, however emphatically Mons. Descuret points to the facts that the Duc de Guise, Louis XVI., and Charles I. were executed in cold weather, and that great revolutions have usually subsided in the heat of summer.

The impetuosity of anger, by exciting the heart and inducing a violent counteraction, may sometimes prove remedial, by raising the mind from the asthenic antipodes of melancholy, and other neuroses. It has suddenly so altered the current of the blood as to cure a paroxysm of gout, (of which cases are recorded by Van Swieten and Haller;) and even, by increase of innervation, to restore the feeling and function of a paralytic limb, as we read in Valerius Maximus and Tulpius.

Revenge, "la crise de la haine," the fatal consummation of the dark passions, is the most Satanic feeling of the human heart. Zanga's demoniac triumph, as he glares on his victim—

> "Groan on, and with the sound refresh my soul,'

and—

> "Vengeance est perdu
> "S'il ignore en mourant que c'est *moi* qui le tue!"

are but the *sentiments* of the wily serpent in Eden. And Collins's lines—

> "With a frown

> Revenge impatient rose;

> He threw his blood-stained sword in thunder down,

> And, with a withering look,

> The war denouncing trumpet took,

> And blew a blast so loud and dread,

> Were ne'er prophetic sounds so full of woe"

might be a very true portraiture of every rebel angel in Pandemonium.

The comparison between the secret assassin and the duellist has often crossed our mind, but we have not space for such digression.

In his chapter on the "Passions of the Insane," (a deeply interesting subject,) Mons. Descuret has some curious tables; one from Pinel, of his nine steps of progress from Reason to Amentia; another showing three double parallel scales, "l'echelle thermometrique," for the degrees of disease, passion, and mania: the bases being health, virtue, and reason, referring to body, soul, and intellect. These passions, however, are but progressive steps of insanity itself, and would involve a complete essay on mental alienation. This chapter will, however, amuse the reader, as will that on the "Passions of Animals."

It would be difficult to form an arrangement of the passions as to positive and negative, active and passive, stimulant and sedative, as this effect must depend, as in the influence of opium, on the degree or dose. Hope exerts a salutary influence within a certain limit, but from intensity or long endurance it may become an excitant. It is therefore in the *regulation* of the passions that a remedial influence is to be sought; or, paradoxical though it appear, in the induction of that sentiment or passion abstractedly prejudicial, that we may thereby antagonize, neutralize, or displace one of an opposite nature already in possession of the mind; as we would administer an *alkali* to render an *acid* inert.

The ensurance of that state of mind which we term repose, tranquillity, contentment,—a state that cannot exist with the debasing passions of pride, envy, hatred, or low ambition,—is itself prophylactic and remedial. Thus old Burton affirms that we cannot "be cur'd till the mind be satisfied;" and Plato, in his allusion to the treatment of Charmides by Socrates, writes, "*nec totum corpus* (*curabis*) *sine animâ.*"

Aided by this anodyne of tranquillity, will the *vis medicatrix* exert its potent influence, and assimilation and other processes proceed healthily. To laugh and grow fat has become a proverb; to ensure this happy mood, how multiform our precepts, moral and physical—amusement, moderate and *congenial* occupation, the bringing of the mind to forego those perilous pleasures of sense, ay, and of *sensibility*, to which luxury and sloth are naturally prone, and to *act on* the subject of its thought, not with fatigue, but that degree of energy that affords food for immediate reflection, the memory of which will be a constant balm to the heart.

To this end there must be both self-control and sympathy of friendship, caution in conversation, allusion to subjects consolatory and congenial, the chamber being adorned with flowers, and objects of interest to the invalid.

In hospital practice, and during the rage of epidemics, we have often witnessed illustrations of the congenial influence of tranquillity. A mild form of primary disorder may prove fatal in an irritable system, while that of very acute degree will run a favourable course where resignation is the characteristic trait.

In the cure of that heart-ache, which, in the words of Lord Bacon, "hath no holidays," the mild precepts of religion will not ill become the lips of the physician, who at least may inculcate prayer and holy patience as an essential item in his catalogue of remedies.

Reflection on the divine precepts, and the adoption of a holy life, may often nip a canker in the bud. If the malady be more advanced, it may still exert its benign influence, when poppy and mandragora will fail; not by harping, like the ill-judging bigot or designing priest, on the hopelessness of a sinner's state; this would increase the evil and drive a penitent to despair; but by assurance of the saving efficacy of repentance and faith—a *waiting* faith in redemption and the promises.

Hope is the most celestial feeling of the mind—the indication of practical confidence in the goodness of the Creator. How beautifully have the poets sung of the charm which hope infuses into the heart. Thus Spencer,—

> " She always smil'd, and in her hand did hold
> A holy water sprinkle dipt in dew,
> With which she sprinkled favors manifold
> On whom she list."

And Collins,—

> " Hope enchanted smil'd, and wav'd her golden hair."

And on her potent spell Campbell has written a bright volume of poesy.

Her steps are over enamelled meads, and her blue eyes are ever turned on the lucid arch of her own congenial heaven.

If hope be confined to earth, it sheds a *couleur de rose* on every thought and thing; and if it soars to heaven while life is ebbing fast, it will whisper peace over the dying couch, and render the sting of death painless. The encouragement of hope, then, is the paramount duty of all who minister to the sick or dying.

There are few feelings more woful than the sudden dashing down of highly-excited hope—it is the fall of a Titan from Olympus to Hades. There is no feeling more delightful than the complete fulfilment of a wish, desire, or longing. The venous trunks that had been loaded during the weight of suspense, the secretion that had been suppressed, instantly feel the removal of the burden, the mind regains its elasticity, and the body has already started in its progress towards health.

Allied to hope, is confidence—a combination of imagination and faith. As an unpropitious announcement will instantly derange the intestinal and gastric functions, so will good news speedily induce healthy action. The sudden entrance of a strange accoucheur will instantly annihilate the parturient effort, which is speedily restored by the arrival of the *engaged* attendant.

The tractors of Perhins, the manipulations of Greatrex, the miracles of Höhenlöhe, are all resulting from this impression. The pious fraud of the Prince of Orange, at the siege of Breda, by which he not only cured the severe scurvy then raging within the walls, but restored heroism to the half-famished soldiers, was based on the influence of imparted confidence.

As a prophylactic, also, confidence is a most potent agent. The immunity of physicians and nurses from infectious disorder depends chiefly on its possession; and we all remember the story of the brave and fearless governor, whose trembling soldiers, commanded to bury the victims of the plague, sunk beneath the pestilence, while *he* survived the duty.

By the way, the thought, as well as the smell or sight, of savoury viands, instantly induces a degree of *salivation*. Might this be rendered remedial as a counteraction or vicarious flow? The brooding over a local malady, too, (imagination and attention,) will often induce, as it will increase, a pain. Might it not thus induce action in a paralyzed muscle?

Joy is a feeling of higher intensity than hope. It is real and active, and centred in the present moment, while hope is quiet and prospective. The joy that springs from the blending of hope and bright memory with present love, is a state on earth little short of Elysium.

The happy influence of joy has been a theme in all ages. In the sacred books of Proverbs and Ecclesiastes, it is written, "A merry heart is the life of the flesh,"—"gladness prolongs his days." Joy is the minimum of that passion which in excess becomes ecstasy. It is hence clear that, like potent physical remedies, even joy must be administered with caution. The Spartan mother fell lifeless at the unexpected return of her son. The felon on the scaffold has dropped dead, as from an electric shock, on the sudden announcement of a pardon! Such was the effect, also, on Chilo the Lacedæmonian, on Sophocles, on Diagoras, and on Leo the Tenth.

Regarding the proximate cause of these fatalities, it would be trifling to discuss the hypotheses of Sanctorius, of Haller, of Cullen, and other pathologists; but it is probable that Haller's notion of *apoplexy* is right. The physical effects of moderate joy are a gentle stimulus to the two great systems, while the ideas assume a character of brightness; the breathing is slightly increased, the bosom gently heaves, the heart beats vigorously, the animal heat is raised, the secretions increased, perspiration ensues, and if the joy becomes intense, tears flow copiously, combined with feelings of extreme gratification.

With this buoyancy of spirit, there is a corresponding elasticity of body; the muscles being stimulated to increased action, which may often be displayed in very whimsical modes, as the *jump for joy.* If, however, this be carried to excess, even phrenitic fever may be lighted up, difficult to manage, and often terminating in mania.

The salutary effect of joy is evident in the freedom of circulation and the healthy performance of the assimilating functions. The liver and pancreas secrete freely, the lacteals are active, the reverse of that induced by the depressing passions. In hypochondriasis and other neuroses sudden and even violent excitement will often be of good effect. Trallianus records the case of a woman who, from the protracted absence of her husband, became maniacal; but on his unexpected return, *instantly* recovered her reason.

Our friend Dr. Uwins records the story of a patient of his, who, during a most stubborn state of hypochondriasis, became completely apathetic. He was taken from one place to another for change of scene; but novelty and exercise did not relieve his malady, until, on his return with his despairing friends, a grotesque figure of a man on horseback excited a most violent and protracted fit of laughter. From that moment, writes our friend, "he was a well man."

The system soon feels the effect of this activity—dyspepsia may be relieved in a few hours. Mons. D. refers, in proof, to the opinions of Hippocrates and Galen, and the many cases recorded by Parè, Tissot, &c. He also tells us that lymphatic engorgements may be soon dissipated by tickling young children. We have seen in our own practice almost an immediate influence of enjoyment. One gentleman, lately under our care, although once of very confined habit, constantly proved the efficacy of the *Times* newspaper about an hour after breakfast; peristaltic action was almost immediately induced. In another gentleman, devoted to topography, the same effect is produced by the perusal of a map, especially of that district with which his mind is interested, or by concentrated reflection on an agreeable excursion. He is an accurate observer, and distinctly feels a thrill of excitement in the course of the splanchnic nerves: and not only is this persuasive stimulus induced, but by a sort of reflex action, his thought and feeling, before apathetic and morose, become active and pleasurable. So, under the influence of joy, intellect becomes brightened, and the mental effort, before made with labour and complaint, becomes an exercise of delight—" labor ipse voluptas."

We may hence learn the importance of adopting modes of amusement *congenial* to the disordered mind—a principle, indeed, now generally observed in the lunatic asylums of our land; and, perhaps, the stimulus of the ruling passion may sometimes be adopted as a remedy, as we administer stimulants in delirium tremens. For instance, the hallooing such words as " quinte, quatorze et le point" into the ears of an insensible or melancholy gambler. Excess of laughter may also effect *mechanically* very extraordinary recoveries. Mons. Descuret relates two cases in illustration. A soldier, apparently dying of a thoracic wound, burst into a violent fit of laughter at a comrade. Instantly two pounds of blood welled forth from the wound, and from that time he gradually recovered. Erasmus also laboured under a vomica which threatened suffocation, but sudden laughter burst the abscess and averted the danger.

Love, sexual love, is a combined passion—a blending of desire and esteem. So there will be often a conflict between the heart and the intellect—the animal inciting to enjoyment, the mental restraining desire. The controlling power of the latter forms the distinction between pure affection and voluptuousness. So, says Burdach—" L'amour de l'homme est plus sensuel, plus jaloux, plus passages, tandis que celui de la femme est plus affectueux, plus confiant, plus fidèle."

But the honey and the sting ever lie close together. There may be but a hair breadth between admiration and passion. Intellect is here the controller of sense. The sense perceives the charm of feminine grace and beauty, the excitement of passion is the natural or instinctive consequence; but this is chastened by intellect, and becomes pure love; the chivalry of the heart and love is no longer animal, but mental.

M. Descuret makes good remarks on the definition, synonymes, causes, character, and symptoms of love, and also on the contrasts of happy, thwarted, or jealous love; the first may be physiological, unless in excess; then, like the other two, it becomes pathological, or disorder. Quaint old Burton and Sir Alexander Crichton have already well analysed these points.

One physical effect of ardent love is probably ever present—acceleration and irregularity of the pulse. We may remember the discovery of the love of Antiochus, the son of Seleuchus, for his mother-in-law, Stratonice, by the acuteness of his physician, Erasistratus. "Ad ejus nomen," writes Plutarch, "rubebat et ad aspectum pulsus variebatur."

The influence of true love is excellent, for it is *unselfish,* and tends to the imparting or diffusion of its own blessings. It is the highest and most beautiful sort of sympathy—

> "That sweet fit that doth true beauty love,
> And chooseth Virtue for his dearest dame."

Like charity, too, it is twice blessed, for it is a pure self-devotion to an object dearer than ourselves. No wonder that a passion, combining the intensest delight of sense with the highest attribute of mind, should be the favourite theme of poets and moralists of all ages. Even its physiology has been deeply studied: these are the beautiful lines of Thomson:—

> "Flush'd by the spirit of the genial year,
> Now from the virgin's cheek a fresher bloom
> Shoots less and less the live carnation round:
> Her lips blush deeper sweets, she breathes of youth,
> The shining moisture swells into her eyes,
> In brighter flow, her wishing bosom heaves
> With palpitations wild; kind tumults seize
> Her veins, and all her yielding soul is love."

If this passion be carried to excess, the throbbing of heart and arteries, the flushing of face and neck, and heat of body, and general erethism, are so much increased as to light up the *fever* of love—"fièvre erotique" of Lorry—thus we have the terms, inflamed by desire, burning with love, and the fire in the heart. Thus the state of love is one replete with sympathies, and not an abstraction of the mind, however the notion of absolute Platonism and the refinements of chivalry *may* suppress the panting for substantial possession and be the relief of an organic impetus.

It would lead us into the delicate discussion of half the maladies of womanhood, were we to comment on the morbid influence of erotic passion, which, in its multiform effect, invades every organ of the body. The "light of love" combines feelings the most delicious that can animate and enliven the frame; but the clouds and shadows of love—fear, envy, jealousy, disappointment, despair—may lead to irremediable evil, or render the system susceptible of every baneful influence.

It is, indeed, mournful to contemplate the ravages which the usages of etiquette and our ultra-refined society are constantly inflicting, even by the "hope deferred" of feminine affection, a full catalogue of which we read in the pages of old Burton. But the anguish of unrequited love in woman, how deep and how intense! A woman's whole life, it is said, is a history of the affections. But let us quote the words of "une femme d'esprit," of whom M. Descuret asked what it was to love: "Pour l'homme c'est être inquiet, pour la femme c'est exister." The heart is the world of woman; it is there her ambition strives for empire: she sends forth her sympathies on a venture; she embarks her whole soul in the traffic of affection; and if shipwrecked, her case is hopeless, for it is a bankruptcy of the heart.

It is this worm i' the bud that so often foils our most anxious study, especially as regards its etiology in chorea, hysteria, melancholia, and other maladies, especially of highly civilized life, and which are far less frequent in the lowly-born and the savage. On these interesting themes M. Descuret presents some very apt illustrations.

Of blighted love, how multiform and sad are the results!

In France, in one year, ninety-four cases of violent death were the result of "*les passions amoureuses*:"—

| | |
|---|---|
| Empoisonnements . . . . . | 23 |
| Assassinats . . . . . . . | 39 |
| Meurtres . . . . . . . | 24 |
| Homicides involuntaire . . . . | 8 |
| | 94 |

The power of beauty over the heart of man is proverbial:—

Νικα δε και σιδηρον
και πυρ καλη τις ουσα.

It has degraded many a Sardanapalus into a slavish voluptuary—an Anthony, who might have been the world's master, into a fool! But as it has thus enervated the hero, it has also raised an idiot to manhood. The story of Cymon and Iphigenia is not a mere fable. And it has done more in the elevation of chivalry, whose noble knights will break their lances and bite the dust for the mere privilege of wearing a lady's favour on their helm, without even a remote dream of passionate indulgence.

But as love's denials prove so detrimental, the happy fulfilment of its promises has averted very serious maladies. If a girl is pale or sad from any of those disorders peculiar to her sex, the mere sentiment of affection will often induce a glow of healthy action, and a change in all the functions. Even incipient phthisis may be averted through the influence of love. But pride, dignity, wealth, and blood will ever raise their scruples against natural affections, if there be disparity of condition—against the natural fulfilment of the Creator's will.

It must be confessed, however, that early romance, and curiosity, and association with vicious domestics, do wofully influence the budding sentiments and actions of girlhood; while the mother keeps aloof, and erroneously reserves those natural secrets which it is her duty delicately to unfold to her daughters.

We must of necessity waive discussion on the "*traitement préservatif et curatif*" of love, and on the subject of aphrodisiacs, &c., to which M. Descuret refers.

Those who are fond of French sentimentality will find some very pathetic stories in this "Manuel," or "Grammaire des Passions," as M. Descuret calls his book of eight hundred pages; and those who have patience and courage to encounter foreign diffuseness, will find a very complete disquisition on the physiology of the passions. We believe that M. Descuret's book may be the foundation of a natural arrangement of mental disorders. In France, it seems to be destined for a still wider range, as we learn that two wise men of very opposite opinions—De Quélen and Broussais—have affirmed that it will become an *essential* part of medical, legal, and theological study.

